# Surgery

**Published:** 1879-11

**Authors:** 


*Collaborators:*

DR. H. GRADLE, DR. L. W. CASE, DR. R. PARK,
DR. R. TILLEY.

### SURGERY.

ANÆSTHETICS.—PRELIMINARY REPORT ON THE ACTION OF ANÆSTHETICS TO THE SCIENTIFIC GRANTS COMMITTEE OF THE BRITISH MEDICAL ASSOCIATION. By a Committee of Joseph Coats, M.D., William Ramsay, PH.D., J. G. McKendrick. (*British Medical Journal, Jan.* 4, 1879.)

Whilst this report is distinctly stated to be merely provisional, it shows clearly the great importance of the work which the committee has undertaken, and the results announced give us one pledge of the benefit physicians are likely to reap from their labors. The comparison of chloroform with ether has clearly demonstrated the former to be decidedly the more dangerous, acting as it does upon the heart to arrest its action, for which there seemed to be little or no relief. Their further investigations have brought to light two other substances which have given them such satisfactory results that they are reserved for complete investigation, namely, the chloride of isobutyl and bichloride of ethidene. Both of these substances produced anæsthesia as quickly as chloroform, and appear to be without its baneful influence on the heart. In experimenting, the heart was exposed so that its action could be distinctly seen. In two such experiments on dogs with the bichloride of ethidene when the anæsthetic was pushed so as intentionally to paralyze the lungs, artificial respiration being maintained, no action of the anæsthetic could be observed on the heart; when, however, chloroform was substituted for the bichloride of ethidene the right side of the heart began almost immediately to distend, get dark and its action rapidly failed. Several curious facts have been noted relative to the effect of small doses of chloroform and ether on the rapidity of nervous and mental processes. It was ascertained that a few respirations of air containing chloroform or ether produced remarkable retardation in the time of signaling back that a visional impression had been perceived, although the person operated upon was quite unconscious of the delay.

The committee has administered the bichloride of ethidene to patients six times with most satisfactory results:

1. The respiration was not disturbed, although anæsthesia was complete.

2. The pulse diminished in frequency and increased in amplitude. When the anæsthesia is complete the pulse is regular, full and compressible.

3. No paleness of the face, no blueness of the lips.

The committee proposes to study further: The action of the chloride of isobutyl on man; the action of anæsthetics on the nerve centers of respiration; the action of chloroform, ether, bichloride of ethidene and the chloride of isobutyl on the blood pressure; and the action of these various substances on protoplasm.

